# 

**Published:** 1915-12

**Authors:** 


### CONTENTS.

| | Page |
|---|---|
| The Foundation of the Medico-Chirurgical Society and The History of Military Medicine in England. By G. Parker, M.A., M.D. Cantab. | 129 |
| Cardiac Diseases and Disorders in Warfare. By Carey Coombs, M.D., M.R.C.P. Lond. | 149 |
| Removal of Bullets and other Metallic Foreign Bodies. By R. G. Poole Lansdown, M.D., B.S. Durham | 157 |
| The Long Fox Lecture: The Evolution of the Sense of Sight. By F. Richardson Cross, M.B. Lond., F.R.C.S. | 163 |
| Gunshot Injuries to the Eyes. By E. H. E. Stack, M.B., F.R.C.S. | 198 |
| Progress of the Medical Sciences: | |
| Surgery | 206 |
| Reviews of Books | 209 |
| Editorial Notes | 218 |
| Meetings of Societies | 226 |
| Obituary Notices | 228 |
| Local Medical Notes | 232 |

